# Struma ovarii with contralateral ovarian teratoma: A case report

**DOI:** 10.3389/fsurg.2022.907326

**Published:** 2022-08-19

**Authors:** Xinjie Ren, Zhaoyou Guo, Jiao Bai

**Affiliations:** Department of Radiology, The Affiliated Hospital of Southwest Medical University, Luzhou, China

**Keywords:** struma ovarii, papillary thyroid cancer, teratoma, tumor ovarii, thyroid carcinoma

## Abstract

Struma ovarii (SO) is a rare form of ovarian teratoma in which the thyroid tissue constitutes over 50% of the tumor. SO comprise 1% of all ovarian tumors. We report the case of a 61-year-old woman who was admitted to the hospital because of frequent urination and dysuria. Abdominal magnetic resonance imaging revealed a mass measuring approximately 16 cm in diameter in the right adnexal area. After transabdominal bilateral adnexectomy, pathological examination revealed a teratoma of the ovary on the right and goiter of the ovary with focal thyroid cancer on the left side. Subsequent total thyroidectomy was performed, and no cancer was found on pathological examination. The patient was treated with thyroxine for a long time after the operation, and there was no recurrence 3 years after diagnosis.

## Introduction

Struma ovarii (SO) is a rare form of ovarian teratoma in which the thyroid tissue forms a grossly detectable mass or is the major cellular component (>50%). Teratomas comprise approximately 95% of germ cell tumors, of which SO accounts for only 3% (1, 2). Tumors are usually benign,but <5% of all SO undergo neoplastic transformation (3–5). The most widespread malignant forms of SO are papillary and follicular carcinomas (6, 7). SO can occur in women of all ages; however, it is most common in women between 40 and 60 years old (7–9). We present the case of a 61-year-old woman who underwent transventral bilateral adnexectomy for bilateral ovarian tumors. After surgery, she was diagnosed with mature teratoma of the right ovary and SO of the left ovary with focal papillary thyroid carcinoma.

## Case presentation

**Figure 1:**
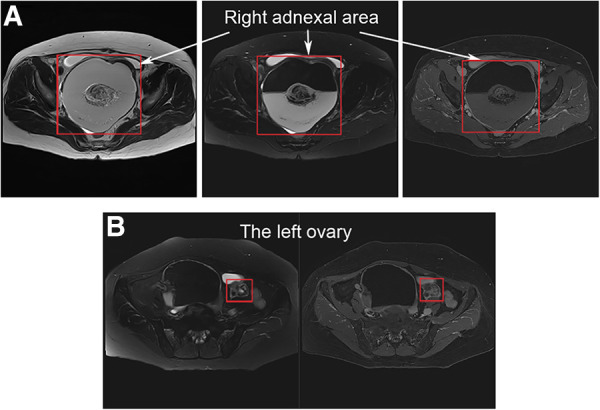
Magnetic resonance imaging of the abdominal cavity and pelvis: (**A**) a vast cystic-solid lesion in the right adnexal area; (**B**) the left ovary is enlarged with multiple vesicular changes.

A 61-year-old woman was admitted to the department of gynecology due to frequent urination, dysuria for 10 + days. Before admission, Doppler Ultrasonography (DU) of pelvic mass and catheterization were performed at another hospital. After catheterization, the patient's symptoms were significantly relieved. Physical examination after admission showed that the patient was in good condition and had stable vital function. A non-tender mass with a diameter of approximately 15 cm, reaching two fingers below the umbilicus, was palpated. DU and magnetic resonance imaging (MRI) of the abdominal cavity and pelvis revealed a cystic mass in the right adnexal area with a long axis of 16 cm (Figure 1). The right ureter and kidney were dilated. Tumor marker levels for Carcinoma Embryonic Antigen and Cancer Antigen 199 were 12.14 ng/ml and 368.70 IU/ml, respectively. The patient underwent transabdominal bilateral adnexectomy. Intraoperative enlargement of the right ovary (approximately 16 × 15 × 15 cm^3^) was observed, presenting multilocular, cystic, and solid changes. The left ovary is enlarged (approximately 4 cm in diameter) with a dark brown surface with multiple vesicular changes. No malignant tumor cells were found in frozen bilateral adnexa during the operation, and the surgical scope was not further expanded.

**Figure 2:**
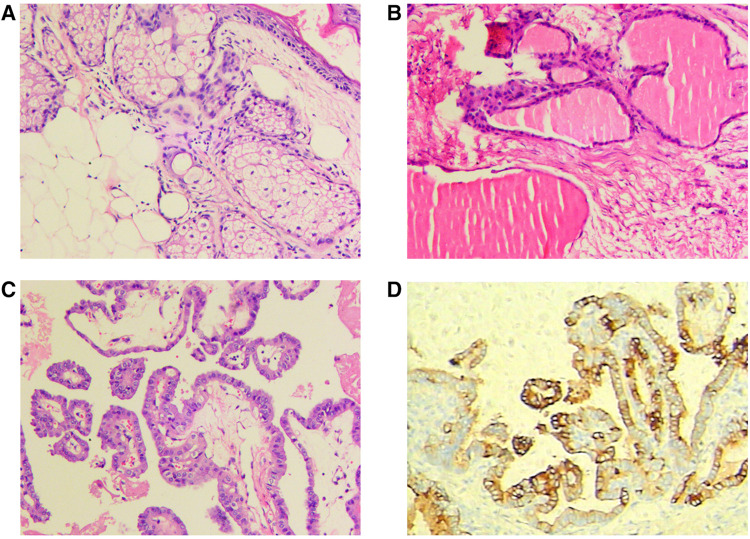
Postoperative pathological examination: (**A**) mature teratoma of the ovary on the right; (**B,C**) goiter of the ovary with active epithelial papillary hyperplasia on the left; (**D**) immunohistochemical examination.

Postoperative pathological examination revealed a mature teratoma of the ovary on the right side and goiter of the ovary with active epithelial papillary hyperplasia on the left side. On morphological and immunohistochemical examinations, a goiter with papillary thyroid carcinoma was considered on the left side (Figure 2).

**Figure 3:**
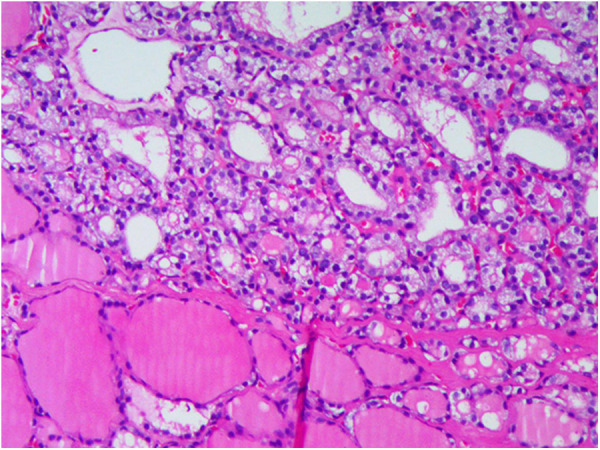
Pathological examination: adenoma in the left thyroid.

Postoperative thyroid DU showed echogenic masses in the right thyroid lobe with calcification and multiple hypoechogenic masses in the left thyroid lobe. The possibility of ovarian metastasis from thyroid cancer cannot be ruled out. No abnormal antibodies to thyrotropin or thyroglobulin were detected. Subsequently, total thyroidectomy and central lymph node dissection were performed. Postoperative pathological examination suggested atypical adenoma with calcification in the right lobe of the thyroid, adenoma in the left thyroid, reactive hyperplasia in some lymph nodes with a few thyroid follicles in one lymph node in the right central region with no obvious atypia, and mostly ectopic thyroid (Figure 3). The patient was treated with long-term thyroxine to reduce thyroid-stimulating hormone (TSH) secretion. Pelvic MRI and neck DU reexamination 9 months after surgery showed no tumor recurrence, and and the patient's condition was uneventful for more than 3 years.

## Discussion

SO can occur in women of all ages; however, it is most common in perimenopausal women and tends to occur in younger women. The clinical presentation of SO is nonspecific and similar to that of other ovarian tumors. The most common symptoms are pelvic tumor, abdominal pain, abdominal distension, abnormal menstrual cycles, vaginal bleeding, frequent and urgent urination, ascites, and deep vein thrombosis (10–12).

In this case, the patient's first symptom involved the urinary tract, which was related to the right teratoma of the ovary that was too large to compress the right ureter.

MRI and DU of SO showed a unilateral cystic and solid mass in the pelvic cavity. Blood flow signals were detected in the solid part on the DU, and thyroid tissue-like enhancement was observed in the MRI-enhanced scan. SO needs to be distinguished from other cystic solid masses, such as: (i) mature teratoma, where fat and calcification are specific and a liquid-liquid plane is visible; (ii) ovarian cystadenoma: compared to SO, the cyst fluid density of ovarian cystadenoma is close to that of water, and the cyst fluid density of SO is higher than that of bladder fluid; (iii) tubal tuberculosis: for patients with pelvic mass, ascites, and cancer antigen 125 < 500 IU/ml, the possibility of tubal tuberculosis needs to be considered (13); and (iv) sex cord stromal cell tumor: as rare as SO and likely to have endocrine function, the coexistence of zoological symptoms and signs of endocrine involvement depends on the secretion of hormones and the origin of the tumor (14).

However, it is generally believed that MRI and DU do not enable the diagnosis of SO, which depends on pathologic examination (10).

At present, the criteria for the pathological diagnosis of SO are (15): (i) the tumor is completely or mainly composed of thyroid tissue (1/2 or more); (ii) thyroid tissue in the mature teratoma forms a generally visible tumor tissue; and (iii) the thyroid tissue in tumors is not as large as 1/2 of the tumor and histologically malignant thyroid tissue, which was combined with the first diagnostic criteria.

SO is generally a nonfunctional tumor; however, many cases suggest that there is a strong relationship between hyperthyroidism and goiter. The following conditions should be considered: (i) the thyroid tissue of the hyperfunctional ovary secretes a large amount of thyroxine; (ii) the thyroid and the ovarian goiter simultaneously produce large amounts of thyroxine; (iii) the thyroid secretes thyroxine and the ovarian thyroid is functional (16). Therefore, in some cases, the cause of hyperthyroidism is not well identified. The patient in this case had no history of hyperthyroidism, had normal thyroid function, and was considered to have a nonfunctional ovarian goiter.

To date, no guidelines have been established for the treatment of thyroid cancer in this location. However, evidence-based recommendations for the management of thyroid cancer were developed by the American Thyroid Association in 2015 (17).

For the treatment of ovarian and thyroid cancer with different recurrence risks, Yassa et al. (6) proposed some treatment plans: (i) for a primary tumor limited to the ovary, smaller than 2 cm, and without aggressive histopathology, they proposed unilateral salpingo-oophorectomy and levothyroxine treatment to maintain serum TSH levels at 0.1–0.5 mIU/L; and (ii) patients with tumors larger than 2 cm or with invasive histopathological features are at high risk and require total thyroidectomy and adjuvant radioactive iodine (I-131) therapy to prevent recurrence. In addition, unilateral oophorectomy can be performed in women with fertility requirements and unilateral disease without capsular invasion or metastases (18).

If ovarian thyroid cancer is associated with distant metastasis, more aggressive treatment (total hysterectomy and bilateral resection of the adnexa and ovaries, omentectomy, total thyroidectomy, and I-131 treatment) is required.

Malignant SO can coexist with thyroid cancer; therefore, we recommend performing total thyroidectomy followed by I-131 treatment to remove possible primary thyroid cancer and micrometastases.

Regardless of the treatment, we recommend at least 10 years of long-term monitoring, including clinical monitoring and thyroglobulin level assessment.

Imaging diagnosis of SO remains difficult. Due to unreliable preoperative and intraoperative diagnoses, histopathological examination must be performed to provide guidance for the treatment and follow-up of patients.

## Data Availability

The original contributions presented in the study are included in the article/Supplementary Material, further inquiries can be directed to the corresponding author/s.

## References

[1] Samina M Wooshin K Arthur RG. Struma ovarii with a focus of papillary thyroid cancer: a case report and review of the literature - ScienceDirect. Gynecol Oncol. (2004) 94(3):835–9. 10.1016/j.ygyno.2004.06.003 15350384

[2] Devaney K. Proliferative and histologically malignant struma ovarii : a clinicopathologic study of 54 cases. Int J Gynecol Pathol. (1993) 12. 10.1097/00004347-199310000-00008 8253550

[3] Mcgill J Sturgeon C Angelos P. Metastatic struma ovarii treated with total thyroidectomy and radioiodine ablation. Endocr Pract. (2009) 15(2):167. 10.4158/EP.15.2.167 19289330

[4] Desimone CP Lele SM Modesitt SC. Malignant struma ovarii: a case report and analysis of cases reported in the literature with focus on survival and I131 therapy. Gynecol Oncol. (2003) 89(3):543–8. 10.1016/S0090-8258(03)00141-0 12798728

[5] Al Hassan MS Tamer S Walid EA Afaf AAA Mahmoud AZ Hanan F The largest reported papillary thyroid carcinoma arising in struma ovarii and metastasis to opposite ovary: case report and review of literature. Thyroid Res. (2018) 11(1):10. 10.1186/s13044-018-0054-9 30061934 PMC6056926

[6] Yassa L Sadow P Marqusee E. Malignant struma ovarii. Nat Clin Pract Endocrinol Metab. (2008) 4(8):469. 10.1038/ncpendmet0887 18560398

[7] Goffredo P Sawka AM Pura J Adam MA Roman SA Sosa JA. Malignant struma ovarii: a population-level analysis of a large series of 68 patients. Thyroid. (2015) 25(2):211–5. 10.1089/thy.2014.0328 25375817

[8] Seung-Chul Y Ki-Hong C Mi-Ok L Chang SJ Hee-Sug R Haeng-So K. Clinical characteristics of struma ovarii. J Gynecol Oncol. (2008) 19(2):135–8. 10.3802/jgo.2008.19.2.135 19471561 PMC2676458

[9] Kraemer B Grischke EM Staebler A Hirides P Rothmund R. Laparoscopic excision of malignant struma ovarii and 1 year follow-up without further treatment. Fertil Steril. (2011) 95(6):2124.e9–2124.e12. 10.1016/j.fertnstert.2010.12.047 21269611

[10] Lamblin G Gallice C Bournaud C Nadaud B Lebail-Carval K Chene GG. Benign struma ovarii: report of 7 cases and review of the literature. Gynecol Obstet Fertil. (2016) 44(5):263–8. 10.1016/j.gyobfe.2016.02.009. 26997461

[11] Xuchen Zhang CA. Thyroid-type carcinoma of struma ovarii. Arch Pathol Lab Med. (2010) 134(5):786–91. 10.5858/134.5.786 20441513

[12] Brusca N Duca SCD Salvatori R D'Agostini A Centanni M. A case report of thyroid carcinoma confined to ovary and concurrently occult in the thyroid: is conservative treatment always advised? Int J Endocrinol Metab. (2015) 13(1):1. 10.5812/ijem.18220 PMC4338667 25745492

[13] Akbulut S Arikanoglu Z Basbug M. Tubercular tubo-ovarian cystic mass mimicking acute appendicitis: a case report. J Med Case Rep. (2011 Aug 10) 5:363. 10.1186/1752-1947-5-363 21831284 PMC3170348

[14] Akbulut S Ceylan SD Tuncali T Sogutcu N. Coexistence of ovarian granulose cell tumor, congenital adrenal hyperplasia, and triple translocation: is a consequence or coincidence? J Gastrointestinal Cancer. (2021 Jun) 52(2):508–14. 10.1007/s12029-020-00408-w 32388791

[15] Wei S Baloch ZW Livolsi VA. Pathology of struma ovarii: a report of 96 cases. Endocr Pathol. (2015) 26(4):342–8. 10.1007/s12022-015-9396-1 26374222

[16] Ang LP Avram AM Lieberman RW Esfandiari NH. Struma ovarii with hyperthyroidism. Clin Nucl Med. (2017) 42(6):475. 10.1097/RLU.0000000000001667 28394842

[17] Chang TC. The application of 2015 American thyroid association management guidelines for adult patients with thyroid nodules and differentiated thyroid cancer in Taiwan and their association with computer-aided detection and diagnosis system. Formos J Endocrinol Metab. (2015) 6(2):53–60. P20180413002-201512-201804130026-201804130026-53-60

[18] Marti JL Clark VE Harper H Chhieng DC Sosa JA Roman SA. Optimal surgical management of well-differentiated thyroid cancer arising in struma ovarii: a series of 4 patients and a review of 53 reported cases. Thyroid. (2012) 22(4):400. 10.1089/thy.2011.0162 22181336

